# Early Phonological Neural Specialization Predicts Later Growth in Word Reading Skills

**DOI:** 10.3389/fnhum.2021.674119

**Published:** 2021-10-14

**Authors:** Brianna L. Yamasaki, Karla K. McGregor, James R. Booth

**Affiliations:** ^1^Department of Psychology and Human Development, Vanderbilt University, Nashville, TN, United States; ^2^Boys Town National Research Hospital, Omaha, NE, United States

**Keywords:** phonological processing, superior temporal gyrus, word reading, reading development, interactive specialization theory, functional magnetic resonance imaging, longitudinal design, Bayesian methods

## Abstract

According to the Interactive Specialization Theory, cognitive skill development is facilitated by a process of neural specialization. In line with this theory, the current study investigated whether neural specialization for phonological and semantic processing at 5-to-6 years old was predictive of growth in word reading skills 2 years later. Specifically, four regression models were estimated in which reading growth was predicted from: (1) an intercept-only model; (2) measures of semantic and phonological neural specialization; (3) performance on semantic and phonological behavioral tasks; or (4) a combination of neural specialization and behavioral performance. Results from the preregistered analyses revealed little evidence in favor of the hypothesis that early semantic and phonological skills are predictive of growth in reading. However, results from the exploratory analyses, which included a larger sample, added age at Time 1 as a covariate, and investigated relative growth in reading, demonstrated decisive evidence that variability in phonological processing is predictive of reading growth. The best fitting model included both measures of specialization within the posterior superior temporal gyrus (pSTG) and behavioral performance. This work provides important evidence in favor of the Interactive Specialization Theory and, more specifically, for the role of phonological neural specialization in the development of early word reading skills.

## Introduction

Contemporary developmental models argue that cognitive skills initially rely on a distributed network of brain regions. Then, over time and practice, that network narrows, as specific regions begin to specialize, and an optimal functional network emerges. According to the Interactive Specialization Theory (Johnson, 2011), it is this process of neural specialization that then facilitates cognitive skill development. Support for this developmental model has been demonstrated across many cognitive domains including face processing, social cognition, and executive control (see Johnson, 2011). Within the linguistic domain, specifically, initial support for this model has come from neuroscientific investigations demonstrating that, for adults, specific linguistic skills are supported by distinct and specialized brain regions (e.g., Price et al., 1997; Poldrack et al., 1999; Devlin et al., 2003; McDermott et al., 2003). Whereas, school-age children show less specialized linguistic networks, as evidenced by both more distributed patterns of activation in response to a variety of linguistic tasks and more similar patterns of activation between linguistic tasks as compared to adults (e.g., Booth et al., 2001, 2003). Furthermore, activation within these language processing regions has been found to be predictive of both concurrent and future linguistic skill in children and adults (e.g., Hoeft et al., 2011; McNorgan et al., 2011; Welcome and Joanisse, 2012; Conant et al., 2014). While this research is supportive of the Interactive Specialization Theory, one limitation to this work is that it has primarily been conducted with individuals who have already established a relatively high level of linguistic proficiency. Much less work has explored the potential for and the role of neural specialization in young children who are still refining their linguistic skills.

The strongest support for neural specialization comes from work demonstrating a double dissociation in which distinct brain regions are found to support one cognitive skill over another (and vice versa). Weiss et al. (2018) conducted one of the first investigations to date using this double-dissociation approach to explore the potential for language-related neural specialization in young children. Specifically, the authors examined whether 5-to-6-year-old children showed evidence of specific brain regions uniquely supporting phonological over semantic processing (or alternatively semantic over phonological processing). In a direct comparison between a phonological judgment task and a semantic judgment task, the authors observed a double-dissociation in which there was greater activity in the left posterior superior temporal gyrus (pSTG) and supramarginal gyrus during phonological processing and greater activity in the left posterior middle temporal gyrus (pMTG) during semantic processing. In addition, specialization-related activity in these regions correlated with performance on relevant behavioral tasks (e.g., phoneme awareness and word association) completed outside of the magnetic resonance imaging (MRI) scanner. The findings from Weiss et al. (2018) provide support that neural specialization begins early in development and that it is related to individual differences in concurrent skill. However, according to the Interactive Specialization Theory (Johnson, 2011), this early neural specialization should not only support current performance but should also facilitate the development of cognitive skills served by phonological and semantic processing.

There is substantial evidence that, among the many cognitive functions they support, early phonological and semantic skills contribute to later reading development (e.g., Catts et al., 1999; Muter et al., 2004; Nation and Snowling, 2004). Much of this research has focused on behavioral predictors, such as performance on various standardized measures. However, researchers within the last few decades have begun to extend this work by exploring language-related brain-based predictors (e.g., McNorgan et al., 2011; Linkersdörfer et al., 2014; Lee et al., 2016; Preston et al., 2016; Smith et al., 2018). A subset of this research, which has examined the use of both behavioral and neural predictors, has shown that both functional- and structural-based neural measures explain unique variance in reading growth over that of purely behavioral measures (e.g., Hoeft et al., 2007, 2011; Maurer et al., 2009; Bach et al., 2013; Myers et al., 2014; Borchers et al., 2019; Jasińska et al., 2020). These studies provide promising support for the predictive utility of neural measures and advance our understanding of the neural bases of reading.

Although previous work has demonstrated the value of both brain- and behavior-based phonological and semantic measures in predicting growth in reading skill, results from this work cannot be used to explicitly test the Interactive Specialization Theory as no studies to date have included direct measures of neural specialization in their models. The aim of the current study was to more directly test the Interactive Specialization Theory by examining whether individual differences in language-related neural specialization predict growth in reading skills over time. In particular, four specific hypotheses were tested. First, consistent with previous research (for a review see Kirby et al., 2008), we hypothesized that individual differences in phonological and semantic processing would predict variability in reading growth, such that better language skills (behaviorally) or more specialized language networks (neurally) at 5-to-6 years old would predict more growth in reading skills. Second, in line with the Interactive Specialization Theory, we hypothesized that measures of neural specialization would be a stronger predictor of reading growth than behavioral measures. Third, given the nature of the outcome variable (i.e., letter and word reading), the young age of the participants, and consistent with behavioral research (e.g., Muter et al., 2004; Schatschneider et al., 2004), we hypothesized that phonological processing would be a stronger predictor of reading growth than semantic processing. Fourth, we hypothesized that any observed relation between neural specialization and reading growth would be driven by both an increase in region-appropriate processing (i.e., phonological processing in pSTG; semantic processing in pMTG) and a decrease in region-inappropriate processing (i.e., semantic processing in pSTG; phonological processing in pMTG).

## Materials and Methods

The study hypotheses, inclusionary criteria, and analytic approach were all pre-registered through the Open Science Framework prior to examining the data or completing the planned analyses.^1^

### Participants

Participants for this study were selected from a larger longitudinal study investigating oral language development in children 5-to-10 years old (Wang et al., submitted). The original sample included 155 children who completed at least part of a functional magnetic resonance imaging (fMRI) session between the ages of 5 and 6.5 years old. The final sample included 30 children who met the preregistered inclusionary criteria (to be described subsequently; 60% of this sample overlapped with the sample used in Weiss et al., 2018). At Time 1 (T1), participants (19 female, 11 male) in the final sample ranged between 5.09 and 6.28 years old (*M* = 5.78). At Time 2 (T2), participants in the final sample ranged between 7.09 and 8.25 years old (*M* = 7.43). An Institutional Review Board approved all study procedures. Before participation, assent and consent were obtained from all participants and their guardians. Participants were compensated with $20 per session, plus an hourly rate of $20/hour, as well as tickets earned over the course of their sessions that could be used to redeem toys and books.

### Procedures

Participants completed developmental questionnaires as well as behavioral and fMRI language and reading tasks over the course of several visits. At T1, participants first completed the questionnaires and standardized language and reading tasks. Following behavioral testing, participants completed 1–2 mock scanner sessions during which the experimenter ensured that they understood the fMRI tasks, they familiarized themselves with the scanner environment, and they practiced the fMRI tasks while in the mock scanner. Finally, participants completed the fMRI tasks in the MRI scanner over the course of 2–4 sessions. At T2, participants completed the standardized reading task.

#### Behavioral Tasks

##### Letter-Word Identification

Performance on the “Letter-Word Identification” subtest of the Woodcock-Johnson Tests of Achievement–3rd Edition (Woodcock et al., 2001) was used to measure individual differences in reading skill. This task was selected given that some participants, particularly at T1, could only identify letters and 2-3 letter words. In this task, participants are asked to verbally identify, visually presented linguistic stimuli of increasing difficulty (e.g., letters to whole words). The total number of correct responses was computed for each participant at T1 and T2. Growth in reading skill, the primary dependent variable in the current study, was operationalized as the difference in performance on the reading task between T2 and T1.

##### Language Tasks

Phonological and semantic processing served as the predictor variables in this study and were measured at T1 both behaviorally and using fMRI. Behaviorally, the “Elision” subtest of the Comprehensive Test of Phonological Processing–2nd Edition (Wagner et al., 1999) was used to measure individual differences in phonological processing. In this task, participants are auditorily presented with a word, asked to repeat the word aloud, mentally remove a phonological segment of that word, and then repeat the new word aloud (e.g., “Say cup, now say cup without saying /k/”). The “Word Classes” subtest of the Clinical Evaluation of Language Fundamentals–5th Edition (Wiig et al., 2003) was used to measure individual differences in semantic processing. On this subtest, participants are presented with a set of items and asked to select the two that match semantically (e.g., visual presentation of a cat, cow, and kitten; “Look, listen, and tell me which two words go together: cat, cow, kitten.”). Raw scores, reflecting the number of correct responses, were used to index individual differences in language processing on each subtest and served as the behavior-based predictors in the analyses.

#### fMRI Tasks

Participants completed two language tasks in the MRI scanner. In both tasks, a trial consisted of two stimuli presented sequentially and binaurally through MRI compatible earphones (Sensimetrics, Model S14). Participants were asked to judge whether the pair of stimuli matched on a given dimension. The right index finger was used for a “yes” response and the right middle finger was used for a “no” response. The duration of each stimulus was manipulated using Praat^2^ to be within the range of approximately 500–700 ms. The first stimulus was presented followed by a pause, which together lasted about 1,000 ms. The second stimulus was presented followed by a jittered response interval. A blue circle was displayed simultaneously with the presentation of the auditory stimuli. The blue circle turned yellow 1,000 ms before the start of the next trial to remind participants to respond if they had not already done so. For each task, participants completed 96 trials, 24 per each of four conditions, divided into two runs. Within each run, trials associated with each of the four conditions were presented in a pseudorandomized order which was consistent across participants.

##### Sound Judgment Task

In the Sound Judgment task, the stimuli consisted of one-syllable words and participants were asked to respond to the question, “Do the two words have any of the same sounds?” There were three experimental conditions: (1) Rhyme, in which the two words shared the same final vowel and phoneme/cluster (corresponding to two-three letters; e.g., WIDE-RIDE); (2) Onset, in which the two words shared the same initial phoneme (corresponding to one letter; e.g., COAT-CAP); and (3) Unrelated, in which the two words shared no phonemes (e.g., ZIP-CONE). All word pairs had no semantic association according to the University of South Florida Free Association Norms (Nelson et al., 2004). There were no significant differences between conditions in word length, number of phonemes, or written word frequency for either the first or second words in a trial within runs (Rhyme vs. Onset: *p*s > 0.173; Rhyme or Onset vs. Unrelated: *p*s > 0.177) or across runs (Rhyme: *p*s > 0.663; Onset: *p*s > 0.436; Unrelated: *p*s > 0.436; linguistic characteristics obtained from the English Lexicon Project, Balota et al., 2007). In addition to the experimental conditions, a control condition was included, in which participants heard pairs of frequency modulated noise sounds and in response were asked to respond with a “yes” button press.

##### Meaning Judgment Task

In the Meaning Judgment task, the stimuli consisted of one- or two-syllable words and participants were asked to respond to the question, “Do the two words go together?” There were three experimental conditions: (1) High Association, in which the words shared a semantic association strength of 0.40–0.85 (*M* = 0.64, SD = 0.13), (2) Low Association, in which the words shared a semantic association strength of 0.14–0.39 (*M* = 0.27, SD = 0.07), and (3) Unrelated, in which the words shared no semantic association. Associative strength was determined based on values from the University of South Florida Free Association Norms (Nelson et al., 2004). There were no significant differences between conditions in word length, the number of phonemes, number of syllables, or written word frequency for either the first or second words in a trial within runs (High vs. Low: *p*s > 0.165; High or Low vs. Unrelated: *p*s > 0.068) or across runs (High: *p*s > 0.162; Low: *p*s > 0.179; Unrelated: *p*s > 0.312; linguistic characteristics obtained from the English Lexicon Project, Balota et al., 2007). In addition to the experimental conditions, a control condition in which participants were asked to press “yes” when they heard a pair of frequency modulated noise sounds was also administered.

#### fMRI Data Acquisition

A 3.0 Tesla Skyra Siemens scanner with a 64-channel head coil was used to acquire all fMRI images. Functional images were acquired using a susceptibility weighted single-shot echo planar imaging (EPI) method with the following parameters: TR = 1,250 ms, TE = 30 ms, multiband acceleration factor = 4, flip angle = 80°, FOV = 256 × 256 mm, voxel size = 2 × 2 × 2 mm, number of slices = 56. A high-resolution T1-weighted structural image was also acquired using the following parameters: TR = 1,900 ms, TE = 2.43 ms, flip angle = 9°, FOV = 256 × 256 mm, voxel size = 1 × 1 × 1 mm, number of slices = 192.

#### fMRI Data Preprocessing and Analysis

MRI data were preprocessed and analyzed using SPM12.^3^ The anatomical images were segmented and warped to a pediatric template. The pediatric template was generated using CereboMatic (Wilke et al., 2017) and a sample of anatomical images acquired on a 3T scanner from 124 children (62 females, 62 males) between the ages of 5.5-to-8 years old. Functional images were first realigned to the mean functional image across runs and then co-registered to the skull-striped anatomical image. Then, functional images were normalized to the pediatric template and smoothed using a 6 mm isotropic Gaussian kernel. Art-Repair^4^ was used to identify outlier volumes among the functional images. Outliers were defined as volumes with greater than 1.5 mm of movement or a 4% deviation in global mean signal intensity volume-to-volume. Interpolated values from adjacent non-outlier volumes were used to replace any volume identified with excess motion or signal deviation.

First-level statistical analyses were performed on individual participants’ data (see Supplementary Table 1 in the Supplementary Materials for a complete list of the specific runs analyzed for each participant) using a general linear model as implemented in SPM12. The first level model included ten regressors for each run, one for each of the experimental conditions (Sound Judgment: Rhyme, Onset, Unrelated or Meaning Judgment: High, Low, Unrelated), one for the control condition, and six nuisance regressors reflecting the realignment parameters. Two contrasts were estimated to index phonological and semantic neural specialization. Phonological specialization was indexed using the contrast [(Onset and Rhyme) − Control] > [(Low and High) − Control]. Similarly, semantic specialization was measured using the contrast [(Low and High) − Control] > [(Onset and Rhyme) − Control].

**Figure 1:**
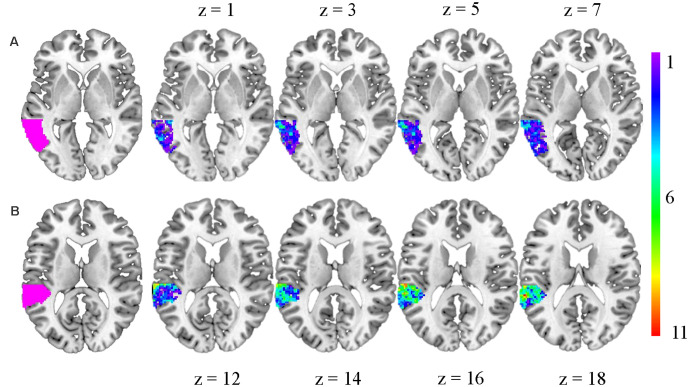
Schematic depiction of the two regions-of-interest (ROIs): **(A)** posterior middle temporal gyrus (pMTG); and **(B)** posterior superior temporal gyrus (pSTG). The first slice in each row represents the anatomical mask within which each participant’s ROI was selected. The color gradient displayed within the other slices represents the spatial overlap among the ROIs across participants. Each color represents the number of participants for which a particular voxel was included within an ROI (see the scale to right).

Following the first-level analysis, individualized regions-of-interest (ROIs) within the pSTG and pMTG were identified for each participant. The pSTG and pMTG were selected as regions of interest based on prior research showing phonological and semantic specialization within these regions, respectively, in both 5-to-6-year-olds (Weiss et al., 2018) and adults (for a review see Binder, 2016). First, an anatomical mask for the left superior temporal gyrus and the left middle temporal gyrus was defined using the automated anatomical labeling (aal) atlas and the WFU pickatlas toolbox (Maldjian et al., 2003). Then, the MarsBar toolbox (Brett et al., 2002) was used to isolate an anatomical mask consisting of the posterior half of the left superior temporal gyrus (pSTG; *y* = −24) and the posterior half of the left middle temporal gyrus (pMTG; *y* = −33). For each participant, the average beta value for each task condition was extracted from the top 100 activated voxels within the pSTG anatomical mask associated with the phonological specialization contrast and the top 100 activated voxels within the pMTG anatomical mask associated with the semantic specialization contrast (see Figure 1). Measures of neural specialization were calculated using these average beta value estimates for each condition in the pSTG and pMTG. These measures served as the brain-based predictors in the analyses.

#### Study Inclusionary Criteria

The following preregistered inclusionary criteria were used to select the final sample for the current study. To be included in the study participants had to:

1. have completed both runs of the Sound Judgment and Meaning Judgment tasks at 5-to-6 years old (with no more than 6-months between runs per task; n excluded = 79)
2. have no more than 10% of total volumes and no more than six consecutive volumes within a run interpolated (n excluded = 11)
3. have obtained adequate performance on the fMRI tasks as indexed by at least 50% accuracy on the control and “easy” (Rhyme and High) conditions and no evidence of response bias (i.e., no greater than a 40% accuracy difference between the Unrelated condition, requiring a “no” response, and the “easy” condition, requiring a “yes” response; n excluded = 27)
4. be primarily right-handed as indexed by performing greater than two out of five tasks (write, draw, turn-over, open, and throw) using their right-hand (n excluded = 0)
5. achieve a standard score of >79 on the Kaufman Brief Intelligence Test–2nd Edition (KBIT-2; Kaufman and Kaufman, 2004; n excluded = 1)
6. have completed the Letter-Word Identification task at 5-to-6 years old and 7-to-8 years old (n excluded = 7)
7. have completed the Elision and Word Classes subtest at 5-to-6 years old (n excluded = 0)

#### Analytic Approach

All analyses were conducted using the lm.beta (Behrendt, 2014), ppcor (Kim, 2015), and BayesFactor (Morey and Rouder, 2018) packages in R (R Core Team, 2019). To evaluate the study hypotheses, four multiple linear regression models were estimated and compared. First, a null, intercept-only, model was estimated (see Formula 1). Then, a Brain model was estimated in which reading growth on the Letter-Word Identification subtest was predicted from measures indexing neural specialization within the pSTG and pMTG (see Formula 2). Next, a Behavior model was estimated in which reading growth was predicted from performance on the Elision and Word Classes subtests (see Formula 3). Finally, a Brain-and-Behavior model was estimated in which reading growth was predicted from both the fMRI- and behavior-based measures of phonological and semantic processing (see Formula 4). Bayes factors (BFs) were used to compare the strength of evidence for each experimental model relative to the null model. Bayesian inference methods were selected to be used in this study as they provide direct evidence for the presence or absence of an effect (i.e., evidence for either the null or the alternative hypothesis). For example, if the relative comparison between the Brain model and the null model resulted in a BF of 10, this would indicate that the observed data were 10 times more probable under the Brain model as compared to the null model. Alternatively, if the comparison resulted in a BF of 0.1, this would indicate that the observed data were 10 times more likely under the null model relative to the Brain model. In the current study, BFs of 10–15 were taken as “strong” evidence, 15–20 were taken as “very strong” evidence, and greater than 20 were taken as “decisive” evidence that the observed data are more probable under the experimental model over the null model (Jeffreys, 1961). Across the experimental models, the best fitting model was determined by evaluating the Bayesian Information Criterion (BIC) values associated with each model, with the best fitting model exhibiting the smallest BIC value. Within each experimental model, semi-partial correlations were used to determine the unique variance explained by each brain or behavior predictor. The significance of the semi-partial correlations was evaluated against a *p* < 0.05 threshold.

**Formula 1 (Null Model)**. *ReadingGrowth*_i_ = *β*_0_ + *ε*_i_

**Formula 2 (Brain Model)**. *ReadingGrowth*_i_ = *β*_0_ + *β*_1_ *Specialization_pSTGi_* + *β*_2_ *Specialization*_pMTGi_ + *ε*_i_

**Formula 3 (Behavior Model)**. *ReadingGrowth*_i_ = *β*_0_ + *β*_1_ *Elision*_i_ +*β*_2_ *WordClasses*_i_ + *ε*_i_

**Formula 4 (Brain-and-Behavior Model)**. *ReadingGrowth*_i_ = *β*_0_ + *β*_1_ *Specialization_pSTGi_* + *β*_2_ *Specialization*_pMTGi_ + *β*_3_ *Elision*_i_ + *β*_4_ *WordClasses*_i_ + *ε*_i_

To evaluate whether an increase in region-appropriate processing or a decrease in region-inappropriate processing was driving any observed relation between neural specialization and reading growth two additional regression models were estimated. Specifically, two contrasts indexing phonological processing [(Onset and Rhyme) > Control] and semantic processing [(Low and High) > Control] were estimated using the average beta values for each condition within the previously identified top 100 activated voxels in the pSTG and pMTG. Estimates based on these contrasts were then used to predict reading growth in two separate regression models. BFs were used to evaluate the model likelihood and BIC values were used to compare model fit across the two language processing models.

## Results

Descriptive statistics for all behavioral and fMRI measures are displayed in Table 1. Overall participants showed a significant increase in reading performance as measured by the Letter-Word Identification task from T1 to T2 (BF > 20.00; *t*_(29)_ = 14.77, *p* < 0.001). Given that the aim of this study was to explore behavioral and neurobiological factors that support growth in reading skill, it is important to note that while there were individual differences in reading skill across both time points the majority of participants were already reading at least some whole words at T1 (e.g., average T1 score = 29; exclusively whole word items on the Letter-Word Identification subtest start on item 15). Paired *t*-tests revealed very strong to decisive evidence that participants performed faster and more accurately on the Rhyme and High conditions as compared to the Onset and Low conditions, thus validating the categorization of the stimuli into the four sets [Sound Judgment task for response times: BF > 20.00, *t*_(29)_ = −3.85, *p* < 0.001; for accuracy: BF > 20.00, *t*_(29)_ = 5.83, *p* < 0.001; Meaning Judgment task for response times: BF > 20.00, *t*_(29)_ = −4.50, *p* < 0.001; for accuracy: BF = 15.78, *t*_(29)_ = 3.34, *p* = 0.002]. However, medium to large correlations were observed between measures of performance on the “Related” conditions within each task, supporting the decision to collapse across the Rhyme/Onset and High/Low conditions for the analyses [Sound Judgment task for response times: BF > 20.00, *r*_(28)_ = 0.86, *p* < 0.001; for accuracy: BF = 8.33, *r*_(28)_ = 0.48, *p* = 0.007; Meaning Judgment task for response times: BF > 20.00, *r*_(28)_ = 0.93, *p* < 0.001; for accuracy: BF > 20.00, *r*_(28)_ = 0.59, *p* < 0.001].

**Table 1:** Descriptive statistics for the behavioral and fMRI tasks (preregistered sample).

			Letter-Word Identification^†^		
	Elision^†^	Word Classes^†^	Time 1	Time 2	Time 2 − Time 1 (Reading Growth)
Mean (SD)	17.4 (6.0)	19.3 (5.2)	29.8 (11.1)	47.3 (8.4)	17.5 (6.5)
Min	9	7	13	29	4
Max	32	26	54	63	29
Max Possible	34	40	76	76
			**Sound Judgment^‡^**		
	**Onset**	**Rhyme**	**Related (Onset and Rhyme)**	**Unrelated**	**Control**
Mean (SD)	1,529 (345)/62.5 (19.2)	1,399 (242)/81.1 (13.8)	1,464 (284)/71.8 (14.3)	1,479 (306)/74.9 (14.2)	1,697 (463)/91.1 (9.7)
Min	1,078/16.7	1,015/50.0	1,068/39.6	1,024/41.7	787/62.5
Max	2,853/95.8	2,254/100.0	2,553/93.8	2,454/100.0	2,550/100.0
			**Meaning Judgment^‡^**		
	**Low**	**High**	**Related (Low and High)**	**Unrelated**	**Control**
Mean (SD)	1,483 (261)/73.6 (16.4)	1,394 (286)/81.7 (9.6)	1,439 (269)/77.6 (11.7)	1,560 (295)/75.7 (15.2)	1,658 (428)/91.0 (7.0)
Min	953/33.3	802/62.5	877/54.2	1,160/33.3	774/70.8
Max	2,235/95.8	2,238/100.0	2,236/97.9	2,270/95.8	2,459/100.0

*Note. † = Raw Scores; ‡ = Response Times (ms)/Accuracy (%)*.

### Preregistered Analyses

Contrary to the predictions motivating the current study, the results from the regression analyses revealed little to no evidence that measures of semantic or phonological processing were predictive of later gains in reading skill [Brain model: BF = 0.56; *F*_(2, 27)_ = 1.76, *p* = 0.191, *R*^2^ = 0.12; Behavior model: BF = 1.08; *F*_(2, 27)_ = 2.79, *p* = 0.079, *R*^2^ = 0.17; Brain-and-Behavior model: BF = 0.68; *F*_(4, 25)_ = 2.10, *p* = 0.111, *R*^2^ = 0.25]. Given that there was little evidence for the experimental models relative to the null model, analyses regarding comparisons between the models (Hypothesis 2), comparisons between the semantic and phonological measures (Hypothesis 3), and follow-up analyses for the brain-based measures (Hypothesis 4) were not conducted.

### Exploratory Analyses

Following the preregistered analyses, a set of exploratory analyses were conducted in which three changes were made to increase the power and specificity of the analyses. First, T1 age was added as a predictor to account for the variability in age observed across participants (see Supplementary Materials for analyses with additional contextual variables). Second, the criteria for behavioral performance were relaxed to allow for the inclusion of more participants. Specifically, all criteria were reduced by 10%, i.e., at least 40% accuracy on the control and “easy” (Rhyme and High) conditions, and no greater than a 50% accuracy difference between the Unrelated and “easy” conditions was required to be included in the exploratory analyses. This change resulted in the inclusion of 10 additional participants (performance on the behavioral and fMRI measures was consistent between samples; see Table 2). Finally, the operationalization of reading growth was adjusted to better reflect growth relative to one’s initial skill level. This was accomplished by dividing each participants’ raw difference score by their initial T1 performance, i.e., (T2 − T1)/T1.

**Table 2:** Descriptive statistics for the behavioral and fMRI tasks (exploratory sample).

			Letter-Word Identification^†^		
	Elision^†^	Word Classes^†^	Time 1	Time 2	(Time 2 − Time 1)/Time 1 (Reading Growth)
Mean (SD)	17.7 (6.0)	18.6 (5.3)	29.4 (10.5)	47.4 (8.3)	0.7 (0.4)
Min	9	7	13	29	0.1
Max	32	26	54	63	1.8
Max Possible	34	40	76	76
			**Sound Judgment^‡^**		
	**Onset**	**Rhyme**	**Related (Onset and Rhyme)**	**Unrelated**	**Control**
Mean (SD)	1,514 (318)/63.6 (18.4)	1,425 (242)/80.0 (14.3)	1,470 (267)/71.8 (14.3)	1,494 (297)/75.0 (13.4)	1,650 (458)/91.7 (8.9)
Min	1,078/16.7	1,015/50.0	1,068/39.6	1,024/41.7	787/62.5
Max	2,853/95.8	2,254/100.0	2,553/95.8	2,454/100.0	2,550/100.0
			**Meaning Judgment^‡^**		
	**Low**	**High**	**Related (Low and High)**	**Unrelated**	**Control**
Mean (SD)	1,480 (240)/75.5 (14.7)	1,405 (267)/82.3 (9.7)	1,443 (246)/78.9 (10.6)	1,542 (277)/72.8 (16.4)	1,640 (428)/91.6 (7.1)
Min	953/33.3	802/58.3	877/54.2	1,160/33.3	774/70.8
Max	2,235/95.8	2,238/100.0	2,236/97.9	2,270/100.0	2,459/100.0

*Note. † = Raw Scores; ‡ = Response Times (ms)/Accuracy (%)*.

**Figure 2:**
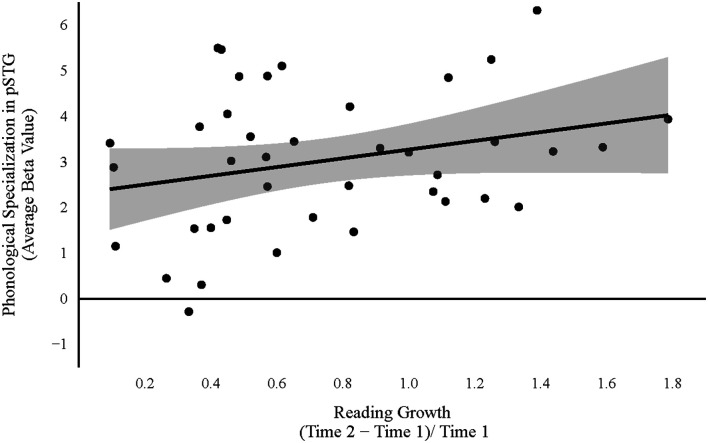
Relation between phonological neural specialization [(Onset and Rhyme) − Control > (High and Low) − Control] in posterior superior temporal gyrus (pSTG) at 5-to-6 years old (Time 1) and growth in reading skill from 5-to-6 years old to 7-to-8 years (Time 2). The shaded region depicts a 95% confidence interval.

**Figure 3:**
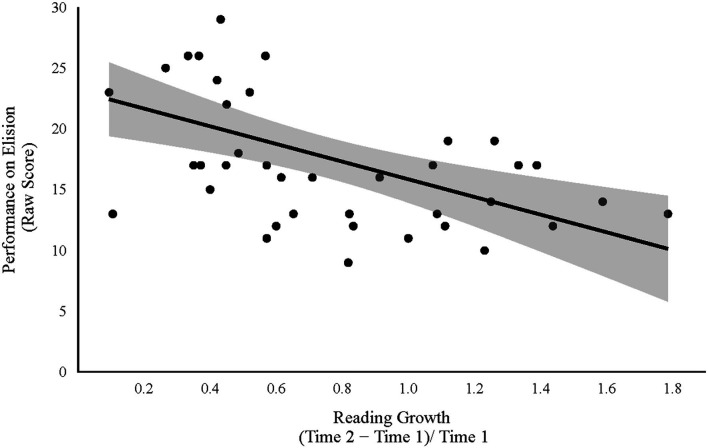
Relation between phonological skill (as measured by performance on the Elision subtest) at 5-to-6 years old (Time 1) and growth in reading skill from 5-to-6 years old to 7-to-8 years old (Time 2). The shaded region depicts a 95% confidence interval.

Consistent with the preregistered analyses, a series of four multiple linear regression models (see Formulas 5–8) were estimated and compared to evaluate the study hypotheses. The regression analysis in which neural specialization was used to predict growth in reading skill resulted in no evidence in favor of the experimental model (BF = 0.51; *F*_(3, 36)_ = 1.95, *p* = 0.139, *R*^2^ = 0.14; BIC = 57.96). Strong evidence was found in favor of a model in which reading growth was predicted from the behavioral measures (BF = 14.77; *F*_(3, 36)_ = 5.63, *p* = 0.003, *R*^2^ = 0.32; BIC = 48.60). However, the strongest evidence was found for the Brain-and-Behavior model, in which growth in reading skill was predicted from both neural specialization and performance on the behavioral measures (BF = 23.68; *F*_(5, 34)_ = 4.95, *p* = 0.002, *R*^2^ = 0.42; BIC = 49.48). Within the Brain-and-Behavior model, only the phonological measures emerged as significant predictors of reading growth (*β*_Elision_ = −0.54, *p* < 0.001; *β*_WordClasses_ = 0.03, *p* = 0.831; *β*_PhonSpec_ = 0.31, *p* = 0.028; *β*_SemSpec_ = −0.04, *p* = 0.789; see Figures 2, 3).

**Formula 5 (Null Model)**. *ReadingGrowth*_i_ = *β*_0_ + *ε*_i_

**Formula 6 (Brain Model)**. *ReadingGrowth*_i_ = *β*_0_ + *β*_1_ *T*1_Age_ + *β*_2_ *Specialization_pSTGi_* + *β*_3_ *Specialization*_pMTGi_ + *ε*_i_

**Formula 7 (Behavior Model)**. *ReadingGrowth*_i_ = *β*_0_ + *β*_1_*T*1_Age_ + *β*_2_ *Elision*_i_ + *β*_3_ *WordClasses*_i_ + *ε*_i_

**Formula 8 (Brain-and-Behavior Model)**. *ReadingGrowth*_i_ = *β*_0_ + *β*_1_ *T*1_Age_ + *β*_2_ *Specialization_pSTGi_* + *β*_3_ *Specialization*_pMTGi_ + *β*_4_ *Elision*_i_ + *β*_5_ *WordClasses*_i_ + *ε*_i_

To examine the robustness of this effect, the analysis was repeated using average beta values estimated from individualized ROIs based on the top 150, top 200, and top 25% of voxels as the brain-based predictors. The results of these additional analyses were directly in line with the exploratory results. That is, across all models, the model with the highest BF was the Brain-and-Behavior model (Top 150: BF_Brain_ = 0.55, BF_Behavior_ = 14.68, BF_Brain-and-Behavior_ = 24.73; Top 200: BF_Brain_ = 0.55, BF_Behavior_ = 14.68, BF_Brain-and-Behavior_ = 23.72; Top 25%: BF_Brain_ = 0.59, BF_Behavior_ = 14.68, BF_Brain-and-Behavior_ = 24.42). Within the Brain-and-Behavior models, only the phonological measures (performance on the Elision subtest and phonological specialization within the pSTG) were significant predictors of reading growth.

**Figure 4:**
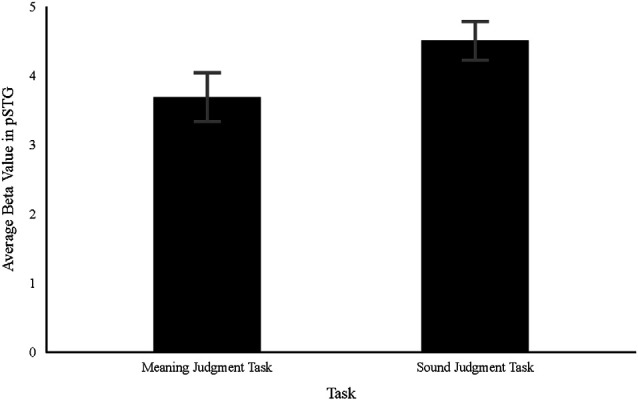
The average beta value within the posterior superior temporal gyrus (pSTG) associated with the Meaning Judgment task [(High and Low) − Control] and the Sound Judgment task [(Rhyme and Onset) − Control]. Error bars depict the standard error of the mean.

Given that there was evidence for the predictive utility of phonological specialization, follow-up regression analyses were conducted to better understand the observed relation between neural specialization and reading growth. Two additional regression models were estimated in which gains in reading were predicted from either average beta value, within the previously identified top 100 activated voxels in the pSTG, associated with the Sound Judgment task [(Onset and Rhyme) > Control] or the Meaning Judgment task [(Low and High) > Control]. Consistent with the other exploratory models, T1 age was included as a predictor in both models. The results of these regression analyses produced little to no evidence for the experimental models [Sound Judgment task: BF = 0.34; *F*_(2, 37)_ = 1.24, *p* = 0.301, *R*^2^ = 0.06; Meaning Judgment task: BF = 0.47; *F*_(2, 37)_ = 1.69, *p* = 0.199, *R*^2^ = 0.08]. An additional analysis was conducted in which the average beta value within the pSTG was estimated for each task individually and then compared. Given that the previously created ROIs were based on a phonological specialization contrast (e.g., Sound Judgment task > Meaning Judgment task), new individualized ROIs were created based on the top 100 activated voxels within the pSTG for each task separately [i.e., Sound Judgment task: (Onset and Rhyme) > Control; Meaning Judgment task: (High and Low) > Control]. Then, average beta values were estimated for each condition within the newly identified ROIs and the task-specific contrasts were re-calculated. A direct comparison between the average beta value estimates for each task revealed strong evidence in favor of the experimental model (in which there is a meaningful difference between the tasks), BF = 13.90; *t*_(39)_ = 3.24, *p* = 0.002. Thus, while individual task activation within the pSTG was not predictive of reading growth, there was a significant difference between the recruitment of the pSTG during the Sound Judgment and Meaning Judgment tasks (see Figure 4).

## Discussion

Results from the preregistered analyses demonstrated little to no evidence that variability in semantic or phonological processing (measured either behaviorally or neurally) reliably predicted individual differences in reading growth. Alternatively, results from the exploratory analyses revealed decisive evidence in favor of the hypothesis that individual differences in early phonological processing will be predictive of later growth in reading skill. Results from the preregistered and exploratory analyses are reviewed in more detail below in light of the four hypotheses that motivated the current study.

### H1: Phonological and Semantic Processing Will Predict Variability in Reading Growth

There is robust evidence that reading is supported by both phonological and semantic skills (for a review see Kirby et al., 2008). Contrary to this work, the preregistered analyses provided no evidence that individual differences in these component skills predicted future gains in reading. However, an examination of the effect sizes for each experimental model revealed that the Brain model (*R*^2^ = 0.12), Behavior model (*R*^2^ = 0.17), and Brain-and-Behavior model (*R*^2^ = 0.25) all explained non-trivial portions of the variance in reading gains.

In the exploratory analyses, the results revealed that the outcome data were ~15–24 times more likely under the Behavior and Brain-and-Behavior models as compared to the null model (BF_Behavior_ = 14.77; BF_Brain-and-Behavior_ = 23.68). This finding suggests that the observed variability in reading growth was more likely to be driven by a model that included measures of language processing than a null model, which included no predictors of interest. While numerically the BIC value associated with the Behavior model was slightly smaller than that of the Brain-and-Behavior model (BIC_Behavior_ = 48.60; BIC_Brain-and-Behavior_ = 49.48), conventional thresholds for evaluating model fit based on BIC values suggests that the difference between the two BIC values is not meaningful (Kass and Raftery, 1995; see Supplementary Materials for analyses with alternative model selection criteria). However, given the substantial difference observed in the magnitude of the BFs associated with the two models (BF_Behavior_ = 14.77; BF_Brain-and-Behavior_ = 23.68), the best fitting model was taken to be the Brain-and-Behavior model, which included both neural and behavioral measures of language processing and explained 42% of the variance in reading gains.

### H2: Neural Specialization Will Be a Stronger Predictor Than Behavioral Measures

The limited existing research in which both behavioral and brain-based measures have been used to predict growth in early reading skills has consistently demonstrated that neural measures explain unique variance over that of behavioral measures (e.g., Hoeft et al., 2007, 2011; Maurer et al., 2009; Bach et al., 2013; Myers et al., 2014; Borchers et al., 2019; Jasińska et al., 2020). In fact, in at least one study, neural measures were found to be predictive of reading growth even when behavioral measures were not (Hoeft et al., 2011). While the preregistered analyses provided little evidence that either brain or behavioral measures of phonological and semantic processing were predictive of readings gains, the results of exploratory analyses revealed decisive evidence in favor of the predictive utility of individual differences in phonological and semantic processing. However, inconsistent with the study hypothesis, a direct comparison between the experimental models revealed that the best fitting model was not the Brain model but instead the Brain-and-Behavior model (BF_Brain_ = 0.51; BF_Behavior_ = 14.77; BF_Brain-and-Behavior_ = 23.68).

The fact that the Brain-and-Behavior model was found to be the best fitting model suggests that in line with previous investigations (e.g., Hoeft et al., 2007, 2011; Maurer et al., 2009; Bach et al., 2013; Myers et al., 2014; Borchers et al., 2019; Jasińska et al., 2020), both neural and behavioral measures explain unique variance in reading growth. However, it should be noted that when evaluating the Brain and Behavior models separately, substantially more evidence was found in favor of the Behavior model as compared to the Brain model. This finding may reflect that the behavioral measures were better able to capture the individual differences in language processing. This would be consistent with the fact that the behavioral measures were normed and standardized measures of language skill as well as the fact that the brain measures focused exclusively on activation within the temporal cortex, which is a critical region for language processing, but only one region within a network of regions known to contribute to language skill (for a review see Binder, 2016).

While both neural and behavioral measures of phonological processing were found to predict individual differences in reading gains, an evaluation of the coefficients associated with each measure suggests that the two measures reflected different relations between the constructs. That is, a positive relation was observed between phonological neural specialization and reading growth (*β*_PhonSpec_ = 0.31), whereas a negative relation was observed between performance on the behavioral measure of phonological skill and reading growth (*β*_Elision_ = −0.54). The positive relation found between phonological neural specialization and reading gains may reflect preliminary evidence for a neural mechanism through which phonological processing facilitates growth in reading skill. Activation within the pSTG during phonological tasks has been associated with lexical access to phonological representations (Graves et al., 2008). Therefore, greater specialization within this region may indicate more refined phonological representations. Consistent with the Interactive Specialization Theory (Johnson, 2011), the positive relation found between specialization and reading growth illustrates that this process of specialization and refinement precedes and facilitates growth in early word reading skills.

The observed negative relation between performance on the behavioral measure of phonological processing and growth in reading skills is inconsistent with what would be predicted based on prior behavioral research. However, there is a critical methodological difference between much of the prior work and the present study that may be an important contributing factor. In particular, much of the previous research showing a positive correlation has explored the longitudinal relation between early phonological skills and later reading skills (e.g., Catts et al., 1999; Muter et al., 2004; Nation and Snowling, 2004) rather than exploring the relation between early phonological skills and growth in reading skills over time. This methodological difference may be driving the observed directional flip in the relation between phonological skill and reading development in the present work. In fact, when Elision performance was correlated directly with T2 Letter-Word Identification performance, a positive relation was indeed observed (BF > 20.00; *r*_(38)_ = 0.76, *p* < 0.001). The negative relation found in the current study likely reflects the fact that those who start with lower phonological (and reading) skills at 5-to-6 years old have more room to improve those skills from 5-to-6 to 7-to-8 years old. This hypothesis is supported by the strong positive relation between Elision and T1 Letter-Word Identification performance (BF > 20.00; *r*_(38)_ = 0.77, *p* < 0.001). An alternative interpretation of this negative relation is that it was driven by the way in which reading growth or language skill was operationalized. In the exploratory analyses reading growth was indexed by dividing each participant’s raw difference score by their initial T1 score. This operationalization allowed for a measure of reading growth that was relative to one’s initial reading performance. However, one consequence of this approach is that those who start with lower T1 reading skills accordingly have smaller values in the denominator of their growth measure, and therefore will show “more” growth than individuals with the same amount of raw growth but higher T1 reading skills. While this operationalization of reading growth did likely contribute to the observed relation, it is not the case that the direction of the relation was driven entirely by this approach as a negative relation was also observed between Elision performance and gains in reading when the exploratory model was estimated with a raw difference score measure of reading growth instead (*β*_Elision_ = −0.29). With regards to the operationalization of language skill, in the current study, phonological and semantic skill were indexed behaviorally through raw scores on the Elision and Word Classes subtests, respectively. While raw scores reflect an individual’s skill level, these scores are not age-adjusted. It is possible that the score profiles and patterns of growth within these skills differ between younger and older participants and that this could have influenced the results observed. To explore this possibility, the exploratory analyses were re-analyzed using scaled scores on the behavioral measures of phonological and semantic skill. The results of this analysis were consistent with the reported findings. That is, the best fitting model was found to be the Brain-and-Behavior model, and, within this model, only the phonological measures were significant predictors of reading growth. Importantly, the results suggest that the use of raw scores did not drive the observed pattern of findings as Elision performance was still found to be negatively associated with reading growth (*β*_Elision_ = −0.55).

### H3: Phonological Processing Will Be a Stronger Predictor Than Semantic Processing

For young children and, in particular, when predicting measures of word reading performance, phonological measures have been found to be a better predictor than semantic measures (e.g., Muter et al., 2004; Schatschneider et al., 2004). Consistent with this work, it was found, in the exploratory analyses, that only phonological predictors significantly explained variance in reading growth. This pattern of results provides support for the broader hypothesis that phonological skills are particularly important for the development of early word reading skills. In addition, it extends previous work by demonstrating that a process of neural specialization within the pSTG may contribute to the relation between phonological processing and reading development. However, given the fact that this finding was only supported by the exploratory analyses, future replications are needed in order to provide more substantial support for the hypothesis that phonological specialization within the pSTG facilitates growth in early reading skills. While variability in semantic processing was not found to be predictive of reading growth in the current study, it is anticipated that semantic processing would emerge as a significant predictor if reading growth was measured in an older group of readers and with measures indexing more advanced reading skill, such as reading comprehension (e.g., Muter et al., 2004).

### H4: Neural Specialization Is Underpinned by Changes in Region Processing

According to the Interactive Specialization Theory, neural specialization should be characterized by both increased engagement during region-appropriate processing and decreased engagement during region-inappropriate processing (Johnson, 2011). Thus, it was predicted that when task-based activity was estimated within the specialized regions and used to predict reading growth, a positive relation would be observed for activity associated with the region-appropriate task and a negative relation would be observed for activity associated with the region-inappropriate task. While a direct comparison between task-based activity within the pSTG showed a significant difference between temporal recruitment during the Sound Judgment vs. Meaning Judgment tasks, the regression analyses relating task-based activity to reading gains provided no evidence in favor of the experimental models. That is, while there was evidence from the exploratory analyses that the degree of differential engagement of the pSTG to the Sound Judgment vs. Meaning Judgment task (i.e., phonological neural specialization) was predictive of reading growth, activity associated with each task individually was not predictive. This finding is inconsistent with both the study predictions and the Interactive Specialization Theory more generally, however it is consistent with the limited previous research on early neural specialization. Weiss et al. (2018) found that performance on phonological and semantic behavioral tasks was correlated with measures of neural specialization but not with task-specific activity in 5-to-6-year-olds. Therefore, what appears to be critical for predicting individual differences in early language and reading skills is not task-specific activity, but rather the degree of differential response patterns in the pSTG to region-appropriate vs. region-inappropriate tasks.

### Limitations and Future Directions

One of the primary limitations in the present study was the lack of converging support between the preregistered and exploratory analyses. The exploratory analyses were, like the preregistered analyses, theoretically motivated, however, the lack of consistency between the analyses weakens the interpretability of the findings. Thus, while the results of the current study provide preliminary evidence for the Interactive Specialization Theory (Johnson, 2011) and, more specifically, for the role of early phonological neural specialization in the development of word reading skills, the data are hypothesis generating rather than confirmatory.

Additionally, one of the central tenets of the Interactive Specialization Theory (Johnson, 2011) is that neural specialization is driven by inter-regional interactions. More specifically, it is hypothesized that over time, variations in regional response biases (e.g., preferential response patterns to particular types of stimuli), experience, and feedback drive regions within a network to interact and compete. These inter-regional interactions ultimately facilitate neural specialization and the development of an optimal functional network. No measures of network-level connectivity were included in the present study and therefore the role of inter-regional interactions could not be tested. However, recent work examining the emergence of the phonological network from 5-to-8-year-olds showed that the strength of connectivity within the phonological network in pre-readers was predictive of later reading skills in emergent readers (Yu et al., 2018). In addition, within the domain of orthographic processing, early connectivity between what will develop into the visual word form area and other brain regions at 5 years old has been found to be predictive of the precise location of this region at 8 years old (Saygin et al., 2016; see also Pleisch et al., 2019). This work provides promising evidence in favor of the interactive component of the Interactive Specialization Theory. However, other studies have shown that while patterns of network-level connectivity do change over development and that the nature of this change is predictive of skill development, these patterns of inter-regional interactions are not necessarily predictive of regional activation within the network (as would be predicted by the Interactive Specialization Theory; Battista et al., 2018). Therefore, future research examining the development of neural specialization and the role of inter-regional interactions is necessary to understand the mechanisms by which such specialization may develop.

Finally, in the current study, each of the experimental models was compared to a null, intercept-only model. While the results of the exploratory analyses suggest that, in line with the Interactive Specialization Theory (Johnson, 2011), individual differences in phonological neural specialization at 5-to-6 years old are predictive of growth in reading skills over time. A more rigorous test of the Interactive Specialization Theory would be to compare a specialization-based brain model to other brain-based models of cognitive skill development. Thus, future investigations should consider extending the current findings by comparing the relative fit of alternative brain-based models as opposed to comparing behavior- and brain-based models, as was done in the present study.

### Conclusion

Understanding the factors that support and constrain reading development is critical given that reading represents a cognitive skill that is highly academically-relevant (e.g., Vineyard and Bailey, 1960) and essential to functioning in modern society (e.g., Raudenbush and Kasim, 1998; DeWalt et al., 2004). Thus, despite the limitations in the present study, the findings from this work provide important information regarding the neural processes that support reading development. Consistent with previous work, the results from the current study provide evidence that individual differences in early phonological processing are an important predictor of later growth in word reading skill. These findings also extend this prior research and provide preliminary support for the Interactive Specialization Theory (Johnson, 2011) by demonstrating that the observed relation between phonological processing and reading growth may be supported by a process of neural specialization for phonological processing within the pSTG.

## Data Availability Statement

The dataset analyzed for this study can be found on OpenNeuro: https://openneuro.org/datasets/ds003604/versions/1.0.2.

## Ethics Statement

This study involving human participants was reviewed and approved by the University of Texas at Austin Institutional Review Board. Written informed consent to participate in this study was provided by the participants’ legal guardian/next of kin.

## Author Contributions

BY conceptualized the project, conducted the formal analysis, wrote the original draft of the manuscript, and created the visualizations. KM reviewed and edited the manuscript. JB acquired the funding for the project and reviewed and edited the manuscript. All authors contributed to the article and approved the submitted version.

## Conflict of Interest

The authors declare that the research was conducted in the absence of any commercial or financial relationships that could be construed as a potential conflict of interest.

## Publisher’s Note

All claims expressed in this article are solely those of the authors and do not necessarily represent those of their affiliated organizations, or those of the publisher, the editors and the reviewers. Any product that may be evaluated in this article, or claim that may be made by its manufacturer, is not guaranteed or endorsed by the publisher.

## Footnotes

^1^Yamasaki, 2019; https://osf.io/ek8rc/

^2^ http://www.fon.hum.uva.nl/praat/

^3^ https://www.fil.ion.ucl.ac.uk/spm/

^4^ http://cibsr.stanford.edu/tools/human-brain-project/artrepair-software.html
