# Supplementary material for: Early Phonological Neural Specialization Predicts Later Growth in Word Reading Skills
Source: Front Hum Neurosci. 2021 Oct 14;15:674119. doi: 10.3389/fnhum.2021.674119 (PMC8551603; doi:10.3389/fnhum.2021.674119)
Supplement: Supplementary file 1 [file Data_Sheet_1.PDF]

Supplementary Materials

1 Supplementary Table

Table 1.
fMRI runs analyzed by participant (pre-registered and exploratory sample)

Participant	Task	fMRI Run	Pre-registered Sample
5008	Phonological	sub-5008_ses-5_task-Phon_acq-D1S10_run-01_bold	X
5008	Phonological	sub-5008_ses-5_task-Phon_acq-D1S12_run-02_bold	X
5008	Semantic	sub-5008_ses-5_task-Sem_acq-D2S5_run-01_bold	X
5008	Semantic	sub-5008_ses-5_task-Sem_acq-D2S3_run-02_bold	X
5010	Phonological	sub-5010_ses-5_task-Phon_acq-D1S12_run-01_bold	X
5010	Phonological	sub-5010_ses-5_task-Phon_acq-D1S14_run-02_bold	X
5010	Semantic	sub-5010_ses-5_task-Sem_acq-D2S7_run-01_bold	X
5010	Semantic	sub-5010_ses-5_task-Sem_acq-D2S9_run-02_bold	X
5015	Phonological	sub-5015_ses-5_task-Phon_acq-D1S7_run-01_bold	X
5015	Phonological	sub-5015_ses-5_task-Phon_acq-D1S15_run-02_bold	X
5015	Semantic	sub-5015_ses-5_task-Sem_acq-D2S5_run-01_bold	X
5015	Semantic	sub-5015_ses-5_task-Sem_acq-D2S11_run-02_bold	X
5023	Phonological	sub-5023_ses-5_task-Phon_acq-D3S3_run-01_bold	X
5023	Phonological	sub-5023_ses-5_task-Phon_acq-D2S5_run-02_bold	X
5023	Semantic	sub-5023_ses-5_task-Sem_acq-D2S14_run-01_bold	X
5023	Semantic	sub-5023_ses-5_task-Sem_acq-D2S12_run-02_bold	X
5024	Phonological	sub-5024_ses-5_task-Phon_acq-D2S7_run-01_bold	X
5024	Phonological	sub-5024_ses-5_task-Phon_acq-D2S5_run-02_bold	X
5024	Semantic	sub-5024_ses-5_task-Sem_acq-D2S19_run-01_bold	X
5024	Semantic	sub-5024_ses-5_task-Sem_acq-D2S17_run-02_bold	X
5029	Phonological	sub-5029_ses-5_task-Phon_acq-D1S12_run-01_bold	X
5029	Phonological	sub-5029_ses-5_task-Phon_acq-D1S14_run-02_bold	X
5029	Semantic	sub-5029_ses-5_task-Sem_acq-D2S12_run-01_bold	X
5029	Semantic	sub-5029_ses-5_task-Sem_acq-D2S14_run-02_bold	X
5034	Phonological	sub-5034_ses-5_task-Phon_acq-D1S7_run-01_bold	X
5034	Phonological	sub-5034_ses-5_task-Phon_acq-D1S9_run-02_bold	X
5034	Semantic	sub-5034_ses-5_task-Sem_acq-D2S7_run-01_bold	X
5034	Semantic	sub-5034_ses-5_task-Sem_acq-D3S15_run-02_bold	X
5054	Phonological	sub-5054_ses-5_task-Phon_acq-D1S4_run-01_bold	X
5054	Phonological	sub-5054_ses-5_task-Phon_acq-D1S6_run-02_bold	X
5054	Semantic	sub-5054_ses-5_task-Sem_acq-D2S7_run-01_bold	X
5054	Semantic	sub-5054_ses-5_task-Sem_acq-D2S5_run-02_bold	X
5055	Phonological	sub-5055_ses-5_task-Phon_acq-D1S8_run-01_bold	X
5055	Phonological	sub-5055_ses-5_task-Phon_acq-D1S10_run-02_bold	X
5055	Semantic	sub-5055_ses-5_task-Sem_acq-D2S9_run-01_bold	X

5055	Semantic	sub-5055_ses-5_task-Sem_acq-D2S7_run-02_bold	X
5061	Phonological	sub-5061_ses-5_task-Phon_acq-D1S4_run-01_bold	X
5061	Phonological	sub-5061_ses-5_task-Phon_acq-D1S7_run-02_bold	X
5061	Semantic	sub-5061_ses-5_task-Sem_acq-D3S3_run-01_bold	X
5061	Semantic	sub-5061_ses-5_task-Sem_acq-D3S4_run-02_bold	X
5070	Phonological	sub-5070_ses-5_task-Phon_acq-D1S7_run-01_bold	X
5070	Phonological	sub-5070_ses-5_task-Phon_acq-D1S5_run-02_bold	X
5070	Semantic	sub-5070_ses-5_task-Sem_acq-D2S9_run-01_bold	X
5070	Semantic	sub-5070_ses-5_task-Sem_acq-D2S7_run-02_bold	X
5071	Phonological	sub-5071_ses-5_task-Phon_acq-D1S20_run-01_bold	X
5071	Phonological	sub-5071_ses-5_task-Phon_acq-D1S10_run-02_bold	X
5071	Semantic	sub-5071_ses-5_task-Sem_acq-D2S5_run-01_bold	X
5071	Semantic	sub-5071_ses-5_task-Sem_acq-D2S3_run-02_bold	X
5091	Phonological	sub-5091_ses-5_task-Phon_acq-D4S20_run-01_bold	X
5091	Phonological	sub-5091_ses-5_task-Phon_acq-D4S22_run-02_bold	X
5091	Semantic	sub-5091_ses-5_task-Sem_acq-D2S5_run-01_bold	X
5091	Semantic	sub-5091_ses-5_task-Sem_acq-D2S3_run-02_bold	X
5118	Phonological	sub-5118_ses-5_task-Phon_acq-D1S6_run-01_bold	X
5118	Phonological	sub-5118_ses-5_task-Phon_acq-D1S4_run-02_bold	X
5118	Semantic	sub-5118_ses-5_task-Sem_acq-D2S3_run-01_bold	X
5118	Semantic	sub-5118_ses-5_task-Sem_acq-D2S5_run-02_bold	X
5126	Phonological	sub-5126_ses-5_task-Phon_acq-D2S20_run-01_bold	X
5126	Phonological	sub-5126_ses-5_task-Phon_acq-D2S18_run-02_bold	X
5126	Semantic	sub-5126_ses-5_task-Sem_acq-D1S8_run-01_bold	X
5126	Semantic	sub-5126_ses-5_task-Sem_acq-D1S6_run-02_bold	X
5140	Phonological	sub-5140_ses-5_task-Phon_acq-D2S3_run-01_bold	X
5140	Phonological	sub-5140_ses-5_task-Phon_acq-D1S12_run-02_bold	X
5140	Semantic	sub-5140_ses-5_task-Sem_acq-D2S12_run-01_bold	X
5140	Semantic	sub-5140_ses-5_task-Sem_acq-D2S14_run-02_bold	X
5159	Phonological	sub-5159_ses-5_task-Phon_acq-D1S33_run-01_bold	X
5159	Phonological	sub-5159_ses-5_task-Phon_acq-D1S23_run-02_bold	X
5159	Semantic	sub-5159_ses-5_task-Sem_acq-D2S11_run-01_bold	X
5159	Semantic	sub-5159_ses-5_task-Sem_acq-D2S9_run-02_bold	X
5160	Phonological	sub-5160_ses-5_task-Phon_acq-D1S9_run-01_bold	X
5160	Phonological	sub-5160_ses-5_task-Phon_acq-D1S7_run-02_bold	X
5160	Semantic	sub-5160_ses-5_task-Sem_acq-D2S12_run-01_bold	X
5160	Semantic	sub-5160_ses-5_task-Sem_acq-D2S10_run-02_bold	X
5199	Phonological	sub-5199_ses-5_task-Phon_acq-D1S3_run-01_bold	X
5199	Phonological	sub-5199_ses-5_task-Phon_acq-D1S5_run-02_bold	X
5199	Semantic	sub-5199_ses-5_task-Sem_acq-D2S10_run-01_bold	X
5199	Semantic	sub-5199_ses-5_task-Sem_acq-D2S8_run-02_bold	X
5215	Phonological	sub-5215_ses-5_task-Phon_acq-D1S11_run-01_bold	X
5215	Phonological	sub-5215_ses-5_task-Phon_acq-D1S9_run-02_bold	X

5215	Semantic	sub-5215_ses-5_task-Sem_acq-D2S2_run-01_bold	X
5215	Semantic	sub-5215_ses-5_task-Sem_acq-D2S4_run-02_bold	X
5242	Phonological	sub-5242_ses-5_task-Phon_acq-D1S13_run-01_bold	X
5242	Phonological	sub-5242_ses-5_task-Phon_acq-D1S3_run-02_bold	X
5242	Semantic	sub-5242_ses-5_task-Sem_acq-D2S6_run-01_bold	X
5242	Semantic	sub-5242_ses-5_task-Sem_acq-D2S8_run-02_bold	X
5244	Phonological	sub-5244_ses-5_task-Phon_acq-D1S9_run-01_bold	X
5244	Phonological	sub-5244_ses-5_task-Phon_acq-D1S11_run-02_bold	X
5244	Semantic	sub-5244_ses-5_task-Sem_acq-D2S6_run-01_bold	X
5244	Semantic	sub-5244_ses-5_task-Sem_acq-D2S8_run-02_bold	X
5259	Phonological	sub-5259_ses-5_task-Phon_acq-D1S7_run-01_bold	X
5259	Phonological	sub-5259_ses-5_task-Phon_acq-D1S9_run-02_bold	X
5259	Semantic	sub-5259_ses-5_task-Sem_acq-D2S5_run-01_bold	X
5259	Semantic	sub-5259_ses-5_task-Sem_acq-D2S12_run-02_bold	X
5267	Phonological	sub-5267_ses-5_task-Phon_acq-D2S20_run-01_bold	X
5267	Phonological	sub-5267_ses-5_task-Phon_acq-D2S15_run-02_bold	X
5267	Semantic	sub-5267_ses-5_task-Sem_acq-D3S14_run-01_bold	X
5267	Semantic	sub-5267_ses-5_task-Sem_acq-D3S12_run-02_bold	X
5270	Phonological	sub-5270_ses-5_task-Phon_acq-D1S5_run-01_bold	X
5270	Phonological	sub-5270_ses-5_task-Phon_acq-D1S3_run-02_bold	X
5270	Semantic	sub-5270_ses-5_task-Sem_acq-D2S8_run-01_bold	X
5270	Semantic	sub-5270_ses-5_task-Sem_acq-D2S6_run-02_bold	X
5286	Phonological	sub-5286_ses-5_task-Phon_acq-D1S3_run-01_bold	X
5286	Phonological	sub-5286_ses-5_task-Phon_acq-D1S5_run-02_bold	X
5286	Semantic	sub-5286_ses-5_task-Sem_acq-D2S2_run-01_bold	X
5286	Semantic	sub-5286_ses-5_task-Sem_acq-D2S4_run-02_bold	X
5304	Phonological	sub-5304_ses-5_task-Phon_acq-D1S8_run-01_bold	X
5304	Phonological	sub-5304_ses-5_task-Phon_acq-D1S3_run-02_bold	X
5304	Semantic	sub-5304_ses-5_task-Sem_acq-D2S5_run-01_bold	X
5304	Semantic	sub-5304_ses-5_task-Sem_acq-D2S7_run-02_bold	X
5336	Phonological	sub-5336_ses-5_task-Phon_acq-D2S4_run-01_bold	X
5336	Phonological	sub-5336_ses-5_task-Phon_acq-D2S2_run-02_bold	X
5336	Semantic	sub-5336_ses-5_task-Sem_acq-D1S3_run-01_bold	X
5336	Semantic	sub-5336_ses-5_task-Sem_acq-D1S5_run-02_bold	X
5338	Phonological	sub-5338_ses-5_task-Phon_acq-D2S5_run-01_bold	X
5338	Phonological	sub-5338_ses-5_task-Phon_acq-D2S3_run-02_bold	X
5338	Semantic	sub-5338_ses-5_task-Sem_acq-D1S7_run-01_bold	X
5338	Semantic	sub-5338_ses-5_task-Sem_acq-D1S9_run-02_bold	X
5352	Phonological	sub-5352_ses-5_task-Phon_acq-D2S6_run-01_bold	X
5352	Phonological	sub-5352_ses-5_task-Phon_acq-D2S8_run-02_bold	X
5352	Semantic	sub-5352_ses-5_task-Sem_acq-D1S7_run-01_bold	X
5352	Semantic	sub-5352_ses-5_task-Sem_acq-D1S11_run-02_bold	X
5009	Phonological	sub-5009_ses-5_task-Phon_acq-D3S5_run-01_bold	
5009	Phonological	sub-5009_ses-5_task-Phon_acq-D3S7_run-02_bold	

5009	Semantic	sub-5009_ses-5_task-Sem_acq-D3S9_run-01_bold
5009	Semantic	sub-5009_ses-5_task-Sem_acq-D3S11_run-02_bold
5045	Phonological	sub-5045_ses-5_task-Phon_acq-D1S10_run-01_bold
5045	Phonological	sub-5045_ses-5_task-Phon_acq-D1S8_run-02_bold
5045	Semantic	sub-5045_ses-5_task-Sem_acq-D2S7_run-01_bold
5045	Semantic	sub-5045_ses-5_task-Sem_acq-D2S5_run-02_bold
5049	Phonological	sub-5049_ses-5_task-Phon_acq-D1S4_run-01_bold
5049	Phonological	sub-5049_ses-5_task-Phon_acq-D1S8_run-02_bold
5049	Semantic	sub-5049_ses-5_task-Sem_acq-D2S9_run-01_bold
5049	Semantic	sub-5049_ses-5_task-Sem_acq-D2S11_run-02_bold
5075	Phonological	sub-5075_ses-5_task-Phon_acq-D1S6_run-01_bold
5075	Phonological	sub-5075_ses-5_task-Phon_acq-D1S8_run-02_bold
5075	Semantic	sub-5075_ses-5_task-Sem_acq-D2S5_run-01_bold
5075	Semantic	sub-5075_ses-5_task-Sem_acq-D2S3_run-02_bold
5077	Phonological	sub-5077_ses-5_task-Phon_acq-D1S8_run-01_bold
5077	Phonological	sub-5077_ses-5_task-Phon_acq-D1S10_run-02_bold
5077	Semantic	sub-5077_ses-5_task-Sem_acq-D2S7_run-01_bold
5077	Semantic	sub-5077_ses-5_task-Sem_acq-D2S9_run-02_bold
5099	Phonological	sub-5099_ses-5_task-Phon_acq-D3S7_run-01_bold
5099	Phonological	sub-5099_ses-5_task-Phon_acq-D3S9_run-02_bold
5099	Semantic	sub-5099_ses-5_task-Sem_acq-D2S12_run-01_bold
5099	Semantic	sub-5099_ses-5_task-Sem_acq-D2S8_run-02_bold
5102	Phonological	sub-5102_ses-5_task-Phon_acq-D1S6_run-01_bold
5102	Phonological	sub-5102_ses-5_task-Phon_acq-D1S4_run-02_bold
5102	Semantic	sub-5102_ses-5_task-Sem_acq-D2S7_run-01_bold
5102	Semantic	sub-5102_ses-5_task-Sem_acq-D2S5_run-02_bold
5109	Phonological	sub-5109_ses-5_task-Phon_acq-D1S10_run-01_bold
5109	Phonological	sub-5109_ses-5_task-Phon_acq-D1S14_run-02_bold
5109	Semantic	sub-5109_ses-5_task-Sem_acq-D2S3_run-01_bold
5109	Semantic	sub-5109_ses-5_task-Sem_acq-D2S5_run-02_bold
5161	Phonological	sub-5161_ses-5_task-Phon_acq-D1S8_run-01_bold
5161	Phonological	sub-5161_ses-5_task-Phon_acq-D1S3_run-02_bold
5161	Semantic	sub-5161_ses-5_task-Sem_acq-D2S2_run-01_bold
5161	Semantic	sub-5161_ses-5_task-Sem_acq-D2S4_run-02_bold
5185	Phonological	sub-5185_ses-5_task-Phon_acq-D1S3_run-01_bold
5185	Phonological	sub-5185_ses-5_task-Phon_acq-D1S5_run-02_bold
5185	Semantic	sub-5185_ses-5_task-Sem_acq-D2S6_run-01_bold
5185	Semantic	sub-5185_ses-5_task-Sem_acq-D2S8_run-02_bold

2 Supplementary Results – Exploring the Role of Contextual Factors

It is possible that other contextual factors related to participants educational, social, and home environments also influence language and literacy development. To more broadly explore the potential influence of these factors, we investigated whether there was a significant relation between

semantic, phonological, or reading skill and a series of child-level factors (i.e., child's age of first spoken word, how long per day child is read to, how long per day child reads on own, how long per day child watches television, and how long per day child uses a computer or tablet) and guardian-level factors (i.e., highest grade level completed by first guardian, highest grade level completed by second guardian, and annual family income). These additional variables were taken from surveys collected as part of the larger study the current sample was selected from. For more information on the procedures for this larger study see Wang et al., submitted and for access to the survey data see https://openneuro.org/datasets/ds003604/versions/1.0.2). For the majority of the additional contextual factors examined, no significant relation was found with any of the language or literacy variables (*p*s = .100 - .963). However, three variables did show significant, or marginally significant, relations. That is, phonological processing skill, as measured by performance on the Elision subtest, was found to vary based on how long per day a child reads (p = .004) and the highest grade level completed by a child's first guardian (p = .024), phonological neural specialization was found to vary based on the highest grade level completed by a child's first guardian (p = .093), semantic processing skill, as measured by performance on the Word Classes subtest, was found to vary based on how long per day a child watches television (p = .050), and reading growth was found to vary based on how long per day a child reads (p = .002). When the exploratory models were re-estimated to include these additional contextual variables, the pattern of results observed was consistent with the reported findings. The best fitting model, showing the lowest BIC value and the highest BF, was found to be the Brain-and-Behavior model (BF = 23.38; $F(8, 31) = 4.23$, p = .002, R^2 = .52). Among the language processing variables, only the phonological predictors were significantly related to variability in reading growth ($\beta_{Elision}$ = -.39, p = .016; $\beta_{PhonSpec}$ = .34, p = .019).

3 Supplementary Results – Model Selection using Akaike Information Criterion Values

In the present study, BIC values were used for model selection however as an additional test of the robustness of the results the exploratory models were reevaluated via the Akaike Information Criterion (AIC), another penalized-likelihood criteria. The pattern of results based on the AIC values was in line with the reported results, that is, the highest AIC value (e.g., lowest model fit) was found for the Brain model (49.51), followed by the Behavior model (40.16) and the Brain-and-Behavior model (37.66).
